# The isotype ZnO/SiC heterojunction prepared by molecular beam epitaxy – A chemical inert interface with significant band discontinuities

**DOI:** 10.1038/srep23106

**Published:** 2016-03-15

**Authors:** Yufeng Zhang, Nanying Lin, Yaping Li, Xiaodan Wang, Huiqiong Wang, Junyong Kang, Regan Wilks, Marcus Bär, Rui Mu

**Affiliations:** 1 College of Physical Science and Technology, Xiamen University (XMU), Xiamen, China; 2 School of Aerospace Engineering, Xiamen University (XMU), Xiamen, China; 3 Renewable Energy, Helmholtz-Zentrum Berlin für Materialien und Energie GmbH (HZB), Berlin, Germany; 4 Energy Materials In-Situ Laboratory Berlin (EMIL), Helmholtz-Zentrum Berlin für Materialien und Energie GmbH, Berlin, Germany; 5 Institut für Physik und Chemie, Brandenburgische Technische Universität Cottbus-Senftenberg, Cottbus, Germany

## Abstract

ZnO/SiC heterojunctions show great potential for various optoelectronic applications (e.g., ultraviolet light emitting diodes, photodetectors, and solar cells). However, the lack of a detailed understanding of the ZnO/SiC interface prevents an efficient and rapid optimization of these devices. Here, intrinsic (but inherently n-type) ZnO were deposited via molecular beam epitaxy on n–type 6H-SiC single crystalline substrates. The chemical and electronic structure of the ZnO/SiC interfaces were characterized by ultraviolet/x-ray photoelectron spectroscopy and x-ray excited Auger electron spectroscopy. In contrast to the ZnO/SiC interface prepared by radio frequency magnetron sputtering, no willemite-like zinc silicate interface species is present at the MBE-ZnO/SiC interface. Furthermore, the valence band offset at the abrupt ZnO/SiC interface is experimentally determined to be (1.2 ± 0.3) eV, suggesting a conduction band offset of approximately 0.8 eV, thus explaining the reported excellent rectifying characteristics of isotype ZnO/SiC heterojunctions. These insights lead to a better comprehension of the ZnO/SiC interface and show that the choice of deposition route might offer a powerful means to tailor the chemical and electronic structures of the ZnO/SiC interface, which can eventually be utilized to optimize related devices.

ZnO is a promising semiconductor material for optoelectronic applications in the ultraviolet (UV) range due to its wide and direct bandgap of 3.4 eV and large exciton binding energy of 60 meV at room temperature^1^,^2^. Although p/n-homojunctions based on ZnO have been fabricated showing promising electroluminescence in the UV wavelength range^3^, preparing p-type ZnO is still challenging (e.g., due to low reproducibility, instability, low conductivity and limited carrier concentration^1^,^2^,^4^,^5^). An alternative approach to make use of the excellent optical properties of ZnO is to employ p/n-heterojunctions that are easy to realize by growing n-type ZnO on suitable p-type semiconductors, such as Al_2_O_3_^6^,^7^, GaN^8^, GaAs^9^, Si^10^, and SiC^11^,^12^. Among these materials, SiC attracts great attention because it is a naturally p-type semiconductor with a bandgap of 3.0 eV (for 6H-SiC)^13^, has a relatively small lattice mismatch to ZnO (4–5% compared to, e.g. 18% between ZnO and Al_2_O_3_)^12^,^14^,^15^, and possesses many additional unique merits (e.g., high thermal stability, excellent chemical stability, high thermal conductivity, and electron mobility)^13^,^16^. It has been shown that SiC and ZnO can form high quality interfaces^17^ promising this material combination to be applied in and beneficial for various optoelectronic devices, such as light emitting diodes, photodetectors, and solar cells. For instance, p-SiC/i-ZnO/n-ZnO heterojunction based diodes have shown random UV lasing^18^; n-ZnO/p-6H–SiC heterojunction based diodes have demonstrated high photoresponsivity to UV light^19^; n-ZnO/n-6H-SiC layer stacks show rectifying characteristics^20^; for ZnO/n-4H-SiC heterojunctions rectification with high breakdown voltage and low leakage current is reported^21^; and the photovoltaic power conversion efficiency of n-ZnO/n-SiC/p-Si heterojunction based solar cells is significantly higher than that of n-ZnO/p-Si based devices^22^.

In order to further improve the performance of related devices, it is necessary to gain detailed information of the chemical and electronic structure of the ZnO/SiC interface. Böhmer *et al.* discovered that SiO_x_ and elemental Zn developed at the ZnO/a-SiC interface when prepared by plasma-enhanced chemical vapor deposition (PECVD), which affects band bending and leads to fill factor losses in related solar cells^23^. Fan *et al.* studied the ZnO/4H-SiC heterojunction prepared by metal-organic chemical vapor deposition (MOCVD). They report an absence of Si-O bonds at the interface, and the valence (VBO) and conduction (CBO) band offsets to be 1.61 eV and 1.50 eV, respectively^14^. Ashrafi *et al.* investigated the ZnO/6H-SiC interface prepared by pulsed laser deposition (PLD), and found a different chemical and electronic interface structure (i.e., Si-O bond formation at the interface, and a VBO of 1.38 eV)^15^,^24^. Overall, these results indicate that the chemical and electronic structures of the ZnO/SiC interface crucially depend on the ZnO preparation route employed and/or the properties of the SiC substrate. Molecular Beam Epitaxy (MBE) is one of the most popular growth techniques, due to its precise control over deposition parameters and *in situ* diagnostic capabilities^1^. However, the chemical and electronic structures of the ZnO/6H–SiC interface prepared by MBE have not been studied in detail. In this paper, we characterize the MBE-ZnO/6H–SiC interface using UV photoelectron (UPS), x-ray photoelectron (XPS) and x-ray excited Auger electron (XAES) spectroscopy and compare our findings to our previous study of the ZnO/6H-SiC interface prepared by radio frequency (RF) magnetron sputtering^25^. We find significant differences in the interface properties that confirm the crucial impact of the ZnO deposition route suggesting an alternative approach for deliberate interface tailoring and device optimization.

## Samples and Instruments

Intrinsic (i.e., prepared without dopants intentionally added) ZnO layers were deposited on n-type 6H-SiC single crystal substrates by MBE. The SiC substrates, purchased from Kejing Materials Technology Co., were cleaned using the RCA cleaning procedure^26^ before transferring them into the MBE deposition chamber (base pressure <5 × 10^−9^ mbar). ZnO layers were deposited using a plasma power of 200 W and 1 × 10^−5^ mbar oxygen partial pressure while keeping the temperature of the Zn source (with a purity of 99.9999%) at 330 °C. A ZnO thickness series was prepared on the cleaned SiC substrates by varying the deposition time using 3, 5, 20, and 30 min. The prepared ZnO/SiC samples were characterized using UPS, XPS, and XAES. These measurements were performed in ultra-high vacuum (base pressure of analysis chamber <2 × 10^−10^ mbar) employing a UV gas discharge lamp (SPECS UVS 10/35) using the He I (21.22 eV) line and a non-monochromatized Mg K_α_ (1253.56 eV) x-ray tube (SPECS XR 50) as excitation sources, respectively. The electrons were detected by a SPECS PHOIBOS 150 MCD-9 electron analyzer calibrated according to procedure described in ref. 27. During the measurements, the samples and the electron analyzer were electrically grounded, and thus all the measurements are recorded with respect to a common Fermi level. The energy scale of the UPS spectra was calibrated using the Fermi level of a clean Au foil. The cleaned SiC substrate and the prepared ZnO thickness sample series were stored and transported under N_2_ inert atmosphere between deposition at XMU and characterization at HZB so that air exposure was minimized.

## Results and Discussion

### Chemical structures of ZnO/SiC interface

Features originating from Si, C, Zn, and O are present in the XPS survey spectra of the cleaned SiC substrate and the ZnO thickness sample series (c.f. Supplementary Information, S.I.-1), as expected. However, O-related (XPS and XAES) signals can also be found on the cleaned SiC substrate, and clear C-related peaks (in contrast to the vanishing Si photoemission lines) can be observed on the ZnO/SiC samples even after 30 min ZnO deposition (about 5.5 nm thick ZnO layer, determined in the next paragraph). This is most likely due to the presence of O- and C-containing surface adsorbates on the studied samples. In the following, we will thus focus on the XPS and XAES features that can exclusively be ascribed to the SiC substrate and ZnO cover layers (i.e., Si 2p and Zn 2p, Zn LMM), respectively.

**Figure 1:**
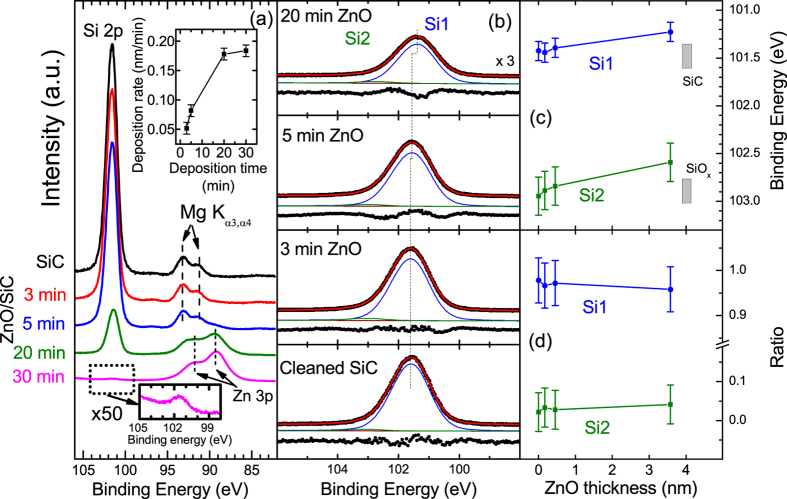
(**a**) Mg K_α_ excited Si 2p and Zn 3p XPS spectra of the cleaned SiC substrate and of ZnO/SiC samples for which ZnO layers have been deposited by MBE onto SiC substrates using different deposition times (3–30 min). The Mg K_α3_ and K_α4_ satellite of the Si 2p photoemission peak that is partially overlapping with the Zn 3p peak is indicated by arrows and dashed lines. The spectra are vertically offset for clarity. Inset: (top) XPS-derived ZnO deposition rate, (bottom) Si 2p signal of the 30 min ZnO/SiC sample enlarged by a factor of 50. (**b**) Fits of the Si 2p XPS spectra of the cleaned SiC substrate and the 3, 5, and 20 min ZnO/SiC samples. For easier comparison, the spectra and respective component fits are vertically offset. Note that the spectrum of the 20 min ZnO/SiC sample is magnified by a factor of ×3. Each fit consists of a linear background and two pairs of Voigt profiles to describe two Si 2p doublets (Si1 and Si2) ascribed to Si in different chemical environments. The magnified (×5) residua (i.e., the difference between data and fit) are also shown. The derived Si 2p_3/2_ binding energy positions of the Si1 and Si2 features and the intensity ratios Si1/(Si1 + Si2) and Si2/(Si1 + Si2) as a function of the XPS-derived ZnO thickness are shown in panels (**c,d**), respectively. The grey boxes in panel (**c**) represent binding energy positions of related reference compounds from refs 31, 32, 33.

The Si 2p and Zn 3p XPS detail spectra of the studied ZnO/SiC samples are shown in Fig. 1(a). The intensity of the Si 2p main peak decreases with increasing ZnO deposition time, due to attenuation by the deposited ZnO layer, as expected. Following the procedure in ref. 28, the thickness (d) of the deposited ZnO layer is derived according to equation (1).


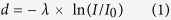


where I_0_ and I are the spectral intensities of Si 2p substrate line before and after ZnO deposition, respectively, and λ is the inelastic mean free path (IMFP) of the Si 2p photoelectrons in ZnO. This model assumes that ZnO layers homogenously cover smooth SiC substrates, and only gives a nominal (i.e., effective) thickness for ZnO layers of other morphology (e.g., island formation). In our case, λ is estimated to be (2.2 ± 0.2) nm (using IMFP values calculated by the QUASES-IMFP-TPP2M code developed by Tougaard^29^), and the (XPS-derived) ZnO deposition rate increases from 0.05 to 0.18 (±0.01) nm/min when increasing the deposition time from 3 to 30 min, as shown in the inset of Fig. 1(a). Note: the Si 2p signal of 30 min ZnO/SiC sample is only visible on a magnified (50×) scale (as shown in the bottom inset of Fig. 1(a)), and suggests that the contribution of the interface in the XPS and XAES spectra of 30 min ZnO/SiC sample can be neglected. Therefore, in the following discussion, the 3, 5, and 20 min ZnO cover layers are considered to be *thin* (in terms of the ability of XPS to probe the interface) and the 30 min ZnO cover layer is considered to be *thick* (as the impact of the interface on the XPS data is minimal).

A curve fit suggests that the Si 2p spectra of the cleaned SiC substrate and the thin ZnO/SiC samples can be well described by two components labeled Si1 and Si2 in Fig. 1(b). Each component was fitted by two Voigt functions with equal Gaussian and Lorentzian widths, constant area ratio (1:2) and energy separation (0.6 eV^30^) to account for the occupancy ratio and spin-orbit splitting of the Si 2p_1/2_, 2p_3/2_ doublet. Based on the influence of varying fitting parameters on the fit result, the uncertainty for the binding energies (BE) of Si1 and Si2 is less than 0.1 and 0.2 eV, respectively. The derived BE of the Si 2p_3/2_ Si1 feature is 101.4 eV before ZnO deposition, and gradually shifts down (up to −0.2 eV) with ZnO deposition. At the same time, the BE of the small Si2 feature changes from 102.9 to 102.6 eV, as shown in Fig. 1(c). Comparing to literature values, the (stronger) Si1 feature is assigned to Si-C bonds^31^ and the (weaker) Si2 feature is associated with Si-O bonds (e.g., SiO_2_ and/or SiO_x_)^32^,^33^. Note that the Si2 feature can also be observed for the cleaned SiC sample which is likely attributed to the presence of a surface oxide indicating the re-oxidation of the SiC substrate after the RCA cleaning or the inability to completely clean the SiC surface. However, the similarity of the BE shifts observed for both Si 2p features with increasing ZnO thickness suggests that the origin of such shifts is an interface-induced band bending, with the bands of the SiC substrate moving upward toward the ZnO/SiC interface. Meanwhile, the energetic position of C-Si feature in the C 1s spectra of the ZnO/SiC samples (shown in Supplementary Information S.I.-2) shifts about the same amount as that of Si features. This further confirms that such shifting is due to an interface-induced band bending. Additionally, within the error bar (±5%) both of the Si2/(Si1 + Si2) and Si1/(Si1 + Si2) intensity ratios do not change with increasing deposition time (see Fig. 1(d)). Note that the slightly increases of Si2/(Si1 + Si2) intensity ratio (from 2% to 4% upon ZnO deposition) implies an abrupt ZnO/SiC interface. This is significantly different from what we observed in our previous study where the interface was formed by RF-sputtering the ZnO on the SiC substrates. In this case, we found the Si2/(Si1 + Si2) intensity ratio to increase from 2% to 10% upon ZnO deposition^25^, which we explained by the RF-sputter deposition induced formation of a zinc silicate – like species at the ZnO/SiC interface^25^. This suggests that the deposition induced chemical interaction involving Si at the ZnO/SiC interface is more significant if the ZnO is deposited by RF sputtering than by MBE.

Since the Si 2p satellite features produced by Mg K_α3_ and K_α4_ excitation of the non-monochromatized X-ray source (8.4 and 10 eV above the main Mg K_α1,2_ energy respectively)^34^ partially overlap with the Zn 3p features and significantly complicate quantification, the Zn 2p and Zn L_3_M_45_M_45_ spectra are examined and discussed below for more detailed (quantified) information of the chemical environment of Zn.

**Figure 2:**
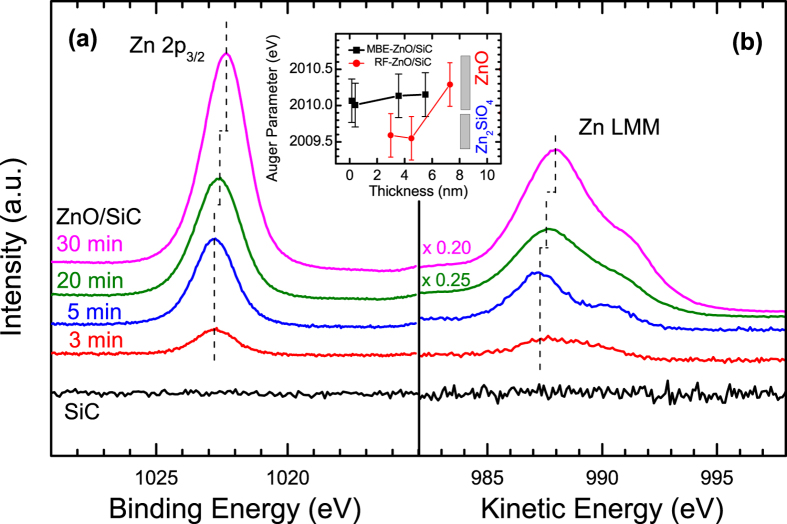
Mg K_α_ excited (**a**) Zn 2p_3/2_ XPS and (**b**) Zn L_3_M_45_M_45_ (LMM) XAES spectra of the cleaned SiC substrate and the ZnO/SiC sample thickness series. The derived modified Auger parameters [AP(Zn) = BE(Zn 2p_3/2_) + KE (Zn L_3_M_45_M_45_)] of the MBE prepared ZnO/SiC samples of this study are compared to the AP(Zn) values of ZnO/SiC samples prepared by RF-sputtering (taken from ref. 25) in the center inset. For comparison, the ranges of reported Auger parameters of reference compounds from refs 38, 39, 40 are also shown.

The modified Auger parameter (AP), which is not influenced by sample charging, doping, or band bending, is the preferred way to characterize chemical species, and can be derived according to the general equation (2):


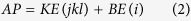


where *BE(i)* is the binding energy of an electron in level *i*, *KE(jkl)* is the kinetic energy of the Auger transition *jkl*^35^. In this study, the BE of the Zn 2p_3/2_ photoemission line, shown in Fig. 2(a), and the KE of the Zn L_3_M_45_M_45_ (Zn LMM) Auger transitions, shown in Fig. 2(b), are used to calculate AP(Zn). The Zn 2p_3/2_ spectra were fit with a linear background and a Voigt function to determine the BE position of Zn 2p_3/2_ line. It changes from (1022.8 ± 0.1) eV for the 3 min ZnO/SiC sample to (1022.4 ± 0.1) eV for the 30 min ZnO/SiC sample. The Zn LMM spectra show a similar shift but in opposite direction, from 987.3 to 987.8 (±0.2) eV KE. Close inspection of the spectra (see Supplementary Information S.I.-3) additionally reveals that the full width at half maximum (FWHM) of the Zn 2p_3/2_ line increases from (1.9 ± 0.1) eV for the 3 and 5 min ZnO/SiC samples to (2.1 ± 0.1) eV for 20 and 30 min ZnO/SiC samples. In parallel, the shape of the Zn LMM spectra changes with ZnO deposition time (see Supplementary Information S.I.-3). Usually this is indicative for Zn being present in more than one chemical environment. However, for the 20 and 30 min ZnO/SiC samples the broadened appearance of the Zn LMM spectra and the wider Zn 2p_3/2_ line might also be an indication for the existence of an electron gas (as expected for high-quality and well conducting ZnO) resulting in additional screening of the core hole and in inelastic scattering of photoelectrons because of excitation of plasmons^36^ as it was previously suggested as an explanation for Zn LMM line shape variations for differently doped ZnO layers by Klein *et al.*^37^. The broad Zn LMM spectrum of the sample with the thinnest ZnO (see also Supplementary Information S.I.-3), can likely be attributed to a less defined material with different bond length and angles. Note that also quantum-size effects might affect the spectral shape in this case – the nominal thickness of the 3 min ZnO/SiC sample is only 0.2 nm, about half of a monolayer of ZnO.

For deriving the modified Auger parameters, we neglected these spectral details and used the BE and KE positions determined assuming that Zn is present in one chemical/electronic configuration. The inset in Fig. 2 shows that within experimental uncertainty, the modified AP(Zn) does not change with deposition time. For example, it is (2010.1 ± 0.3) eV for the 3 min ZnO/SiC sample, and (2010.0 ± 0.3) eV for the 30 min ZnO/SiC sample. These values are consistent with reported modified APs(Zn) for ZnO, which range from 2009.8 to 2010.5 eV^38^,^39^, and differ from the APs(Zn) of zinc silicates^40^ – see grey boxes in the inset of Fig. 2. Apparently, the interface formed when ZnO is deposited by MBE on SiC is chemically abrupt. This result is – as pointed out above – significantly different from the result of our previous study of the ZnO/SiC interface prepared by RF magnetron sputtering^25^. In the study, the Ap(Zn) changed from 2009.6 to 2010.9 eV with increasing ZnO deposition time (see red data points in the inset of Fig. 2), indicating a significant chemical interaction at the RF-ZnO/SiC interface that we interpreted as the formation of an interlayer of willemite-like zinc silicate^25^. Thus, in addition to preparation parameters, the choice of the deposition route might offer an alternative means to tailor – by the presence or absence of a Zn_2_SiO_4_-like dielectric interlayer – the chemical (and thus also the electronic) structure of the ZnO/SiC interface. The ability to form (or avoid) an interface layer with a large band gap (E_g_ (Zn_2_SiO_4_) > 5 eV)^41^ could be easily exploited to deliberately introduce a chemical passivation layer of optimal thickness at the ZnO/SiC interface.

### Electronic structure of ZnO/SiC interface

**Figure 3:**
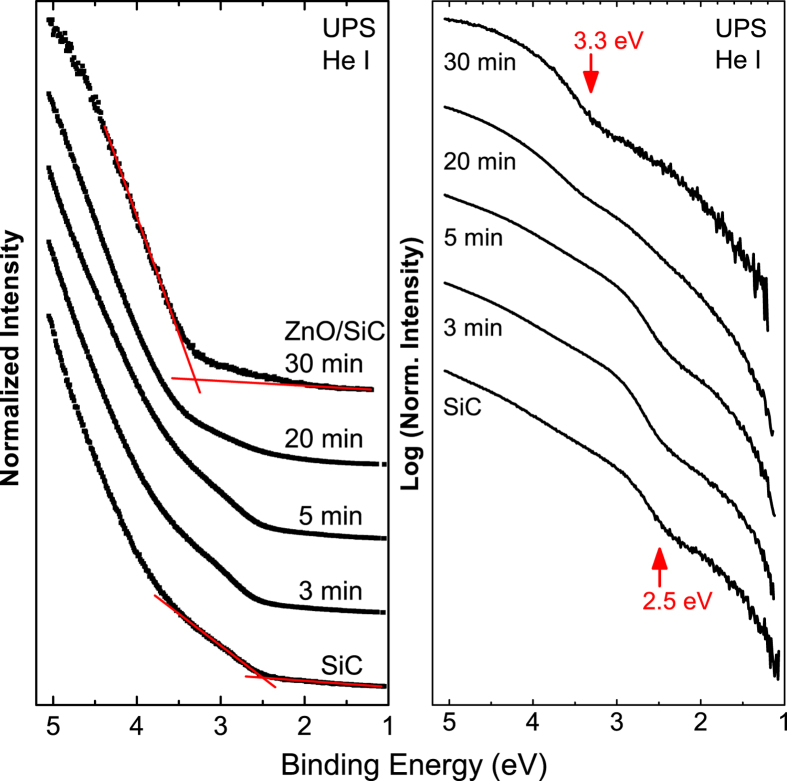
He I excited UPS valence band spectra of the cleaned SiC substrate and the ZnO/SiC samples, with different thickness ZnO layer as indicated by the MBE deposition times 3, 5, 20, and 30 min. The spectra are shown on a linear (left) and logarithmic (right) scale. The red lines in the left panel indicate the linear extrapolation of the leading UPS edge to the base line to derive the position of the valence band maximum (VBM) with respect to the Fermi level. The thus derived VBM values are also indicated by arrows in the right panel and have an error bar of ±0.1 eV for the SiC sample and ±0.2 eV for the 30 min ZnO/SiC sample.

Next, we focus on the electronic structures of the ZnO/SiC interface aiming at the determination of a complete band alignment picture. The valence band (VB) UPS spectra of the cleaned SiC substrate and the ZnO/SiC sample thickness series are shown in Fig. 3 on linear (left panel) and logarithmic (right panel) scale. Both presentations clearly show that the VB density of states (DOS) gradually change from indication of SiC to that of ZnO with increasing MBE deposition time, i.e. ZnO thickness. The spectral intensity in the range between 2 and 4 eV below the Fermi level (E_F_) can thus be associated with the VB DOS of SiC, while the intensity between 3 and 5 eV below E_F_ can be ascribed to the ZnO VB DOS. The spectra of the 3 and 5 min ZnO/SiC samples are clearly dominated by the SiC VB DOS and the spectrum of the 30 min ZnO/SiC sample mainly shows intensity related to the VB DOS of ZnO. This agrees with the intensity evolution of the Si 2p feature (it decreases with increasing ZnO deposition time as shown in Fig. 1) and Zn 2p feature (it increases with increasing ZnO deposition time as shown in Fig. 2). The 20 min ZnO/SiC sample represents a borderline case: Its UPS spectrum unambiguously shows intensity that can be attributed to SiC as well as to ZnO VB DOS. However, we deliberately refrain from using this spectrum to directly derive the VB offset as it is not straightforward to separate the spectral intensities attributed to ZnO and SiC VB DOS. (One complication is the pronounced “foot” that can be observed in the ZnO dominated UPS spectrum of the 30 min ZnO/SiC sample that overlaps with spectral VB contributions from SiC – a discussion of this pre-edge feature can be found below.) Instead, we are using the positions of the VB maxima (VBM) of the cleaned SiC and the *thick* (i.e., 30 min) ZnO layer together with the interface-induced band bending as derived by monitoring the core level shifts using the *thin* (i.e., 3, 5, 20 min) ZnO/SiC samples.

First, we derive the VBM positions for SiC and ZnO by linear extrapolation of the leading edge in the UPS spectra (see red lines in Fig. 3, left panel). Note that we assume the spectral VB contributions from O- and C-containing surface adsorbates (see above) to not dominate this spectral region, as O- and C-derived VB contributions are expected to appear further away from E_F_. The thus derived VBM value for the cleaned SiC sample is (2.5 ± 0.1) eV – as also indicated by an arrow in Fig. 3 (right panel). This value is similar to reported values^15^,^24^, indicating that in our case surface contaminants indeed have at most a minor impact on the VBM determination. Therefore, we did not employ additional cleaning procedures (e.g., sputtering), which often lead to an artificially altered surface, for the ZnO/SiC samples before UPS measurements. However, the significant spectral intensity right at the onset of the UPS spectrum (see Fig. 3 and above) makes it difficult to use the linear extrapolation approach to derive the VBM of the 30 min ZnO/SiC sample. A similar pre-edge intensity was observed before for RF-sputtered ZnO^42^. This can be indicative for the fundamental problem of determining the correct (k-resolved) VBM with He I excited UV photoemission, due to a potential lack of suitable photoemission final states at the Γ point (see, e.g. ref. 43 and refs therein.) and/or defect-induced emission, as is well known for highly doped semiconductors^44^. In order to check whether the “foot” hides the *true* VBM, the UPS data is presented on a logarithmic scale in Fig. 3 (right panel). However – within the signal-to-noise ratio of our data – we do not find evidence for this. Consequently, we used a linear extrapolation of the leading UPS edge as shown in Fig. 3 (left panel) to determine the VBM of the 30 min ZnO/SiC sample. We find a value of (3.3 ± 0.2) eV – as also depicted by the arrow in Fig. 3 (right panel). Note the larger error bar compared to the VBM value of the SiC sample that accounts for the larger uncertainty related to the pre-edge feature and the applicability of the linear extrapolation approach discussed above. In any case, the determined VBM is in good agreement with previously reported values^1^,^15^,^24^,^44^.

Based on the derived VBM positions for SiC (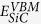) and ZnO (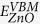) the valence band offset (*E*_*VBO*_) at the ZnO/SiC interface can now be calculated by equation (3) taking the interface-induced band bending into account:


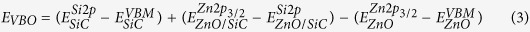


where 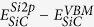 is the energy difference between the position of the VBM and the Si 2p core level of the cleaned SiC; 
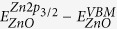
 is the energy difference between the VBM and the Zn 2p_3/2_ core level of the thick (i.e., 30 min) ZnO layer; and 
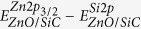
 is the energy difference between the Si 2p and Zn 2p_3/2_ core levels of the thin (i.e., 3, 5, 20 min) ZnO/SiC samples.

**Figure 4:**
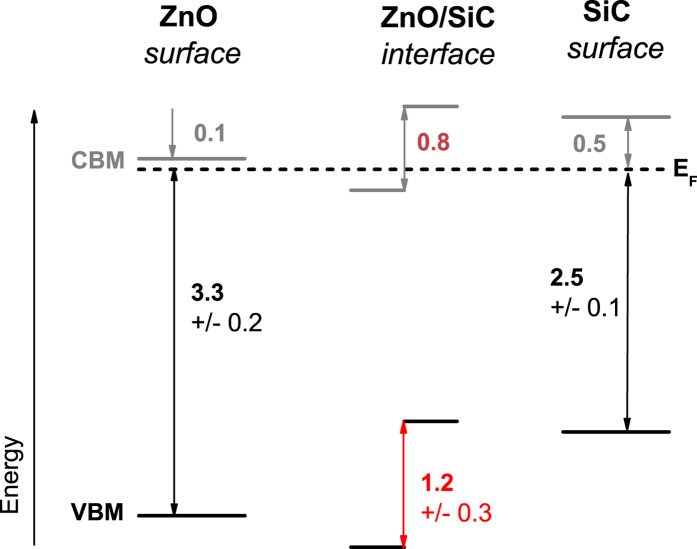
Schematic presentation of the band alignment at the ZnO/SiC interface. The left and right schemes show the position of the energy levels for the ZnO and SiC *surface*, respectively. The center panel visualized the band alignment at the ZnO/SiC *interface* considering interface-induced band bending. The energy positions related to the valence band are directly measured by XPS and UPS while the conduction band positions are estimated taking the materials’ band gap values from ref. 1, 2, 12 and 44 into account. The unit for all numbers is eV.

Using the determined BE of the Si 2p_3/2_ (Si1) feature of (101.4 ± 0.1) eV and the respective VBM value of (2.5 ± 0.1) eV for the cleaned SiC substrate, we derive an energy difference 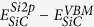 of 98.9 eV. Similarly, we use the BE of the Zn 2p_3/2_ line [(1022.4 ± 0.1) eV] and the corresponding VBM position [(3.3 ± 0.2) eV] to compute an energy difference 
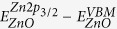
 of 1019.1 eV for the thick (30 min) ZnO/SiC sample. Furthermore, the energetic position of the Si 2p_3/2_ (Si1) and Zn 2p_3/2_ core levels of the thin ZnO/SiC sample is (101.3 ± 0.1) eV and (1022.7 ± 0.1) eV, respectively. Note: these values represent the average BE positions for all three thin ZnO/SiC samples. Hence, the energy difference of the two core levels is 921.4 eV. With that, the VBO is calculated to be (1.2 ± 0.3) eV, as visualized in the band alignment scheme shown in Fig. 4. Based on this measured VBO also the CBO (*E*_*CBO*_) can be estimated by considering the difference in the (bulk) bandgap of SiC (more exact: 6H-SiC) 
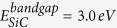
13,45 and ZnO 
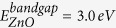
1,2:


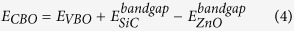


Using equation (4), the CBO at the ZnO/SiC interface is approximated to be 0.8 eV (see Fig. 4). Note that this approach assumes that the (bulk) band gaps of the involved materials also represent the optoelectronic properties of the material surface and heterointerface, respectively. Furthermore, as we use experimentally derived bulk band gap values taken from literature that does not provide any information about the experimental uncertainty^1^,^2^,^12^,^44^, we do consider the stated CBO value as a coarse estimate and do not give an error bar. However, comparing the derived VB and CB offsets with literature we find that they are significantly smaller than the VBO and CBO values reported for the ZnO/4H-SiC interface prepared by MOCVD that showed no evidence for deposition-induced interfacial Si-O bond formation^14^. But they do somewhat agree – within the experimental uncertainty – with the offsets found for the ZnO/6H-SiC system that has been prepared by PLD that did show Si-O bond formation at the interface)^24^ – see comparison in Table 1.

This indicates that there is a strong correlation between chemical and electronic interface properties, which is significantly influenced by the employed deposition technique, order of deposition, and the chemical and electronic structures of the heterojunction partners. Based on the band alignment shown in Fig. 4, we speculate that the significant offsets in the valence as well as the conduction band prevent efficient charge carrier transport across the n-type ZnO/n-type 6H-SiC interface. In conclusion, although somewhat contradicting the energy band diagram suggested in ref. 20, this might be the reason for the reported excellent rectifying characteristics of isotype ZnO/SiC heterojunctions^20^,^21^. The fact that just by choice of the deposition method it is possible to introduce a passivating zinc silicate – like dielectric layer with a large band gap at the ZnO/SiC interface might also be responsible for the reported high breakdown voltages and low leakage currents^21^.

## Conclusion

Intrinsic (but inherently n-type) ZnO layers of different thicknesses were deposited on n-type 6H-SiC single crystals by MBE. The samples were characterized using x-ray photoelectron and Auger electron spectroscopies. A detailed analysis of the evolution of the Si 2p, Zn 2p, and Zn L_3_M_45_M_45_ spectra with increasing ZnO thickness indicates that no significant chemical interaction takes place at the MBE-ZnO/SiC interface, which is dramatically different from the ZnO/SiC interface prepared by RF magnetron sputtering, where we observed the formation of a willemite-like zinc silicate interface species^25^ that could act as a chemical passivation layer at the ZnO/SiC interface. Together with the significant offsets in the valence band (*measured* VBO = 1.2 ± 0.3 eV) and conduction band (*estimated* CBO ≈ 0.8 eV) derived for the ZnO/SiC interface, this might explain the excellent rectifying characteristics of isotype ZnO/SiC heterojunctions. Thus, just by a deliberate choice of the ZnO deposition method, it seems feasible to tailor the chemical (and thus also the electronic) structures of the ZnO/SiC interface, and open an easy optimization route for ZnO/SiC – based optoelectronic devices.

## Additional Information

**How to cite this article**: Zhang, Y. *et al.* The isotype ZnO/SiC heterojunction prepared by molecular beam epitaxy – A chemical inert interface with significant band discontinuities. *Sci. Rep.* **6**, 23106; doi: 10.1038/srep23106 (2016).

## Supplementary Material

Supplementary Information

## Figures and Tables

**Table 1:** The valence and conduction band offsets at differently prepared ZnO/SiC interfaces.

Interface	VBO (eV)	CBO (eV)	Reference
ZnO/6H-SiC	1.2 ± 0.3	*0.8*	this study
ZnO/6H-SiC	1.38 ± 0.28	1.01 ± 0.28	A. Ashrafi ref. 24
ZnO/4H-SiC	1.61 ± 0.23	1.50 ± 0.23	H. Fan ref. 14

*Italic* numbers indicate estimated values (see text for more details)
