# Supplementary material for: The isotype ZnO/SiC heterojunction prepared by molecular beam epitaxy – A chemical inert interface with significant band discontinuities
Source: Sci Rep. 2016 Mar 15;6:23106. doi: 10.1038/srep23106 (PMC4791549; doi:10.1038/srep23106)
Supplement: Supplementary Information [file srep23106-s1.pdf]

SUPPLEMENTARY INFORMATION

The isotype ZnO/SiC heterojunction prepared by molecular beam epitaxy – A chemical inert interface with significant band discontinuities

Yufeng Zhang[1], Nanying Lin[1], Yaping Li[1], Xiaodan Wang[1], Huiqiong Wang[1], Junyong Kang[1], Regan Wilks[3,4], Marcus Bär[3,4,5], and Rui Mu[2*]

1. College of Physical Science and Technology, Xiamen University (XMU), Xiamen, China
2. School of Aerospace Engineering, Xiamen University (XMU), Xiamen, China
3. Renewable Energy, Helmholtz-Zentrum Berlin für Materialien und Energie GmbH (HZB), Berlin, Germany
4. Energy Materials In-Situ Laboratory Berlin (EMIL), Helmholtz-Zentrum Berlin für Materialien und Energie GmbH, Berlin, Germany
5. Institut für Physik und Chemie, Brandenburgische Technische Universität Cottbus-Senftenberg, Cottbus, Germany

*Corresponding author: murui@xmu.edu.cn

The XPS survey spectra of the cleaned SiC substrate and the ZnO/SiC thickness series prepared by MBE with varying deposition time are shown in S.I.-1. The spectra consist of features related to Si, C, Zn, and O species, as expected. However, O signal can be observed in the spectra of the cleaned SiC, and unlike the Si lines the C features do not completely disappear even in the thickest (i.e., 30 min) ZnO/SiC sample. This indicates that the existence of C and O related surface absorbates in the samples.

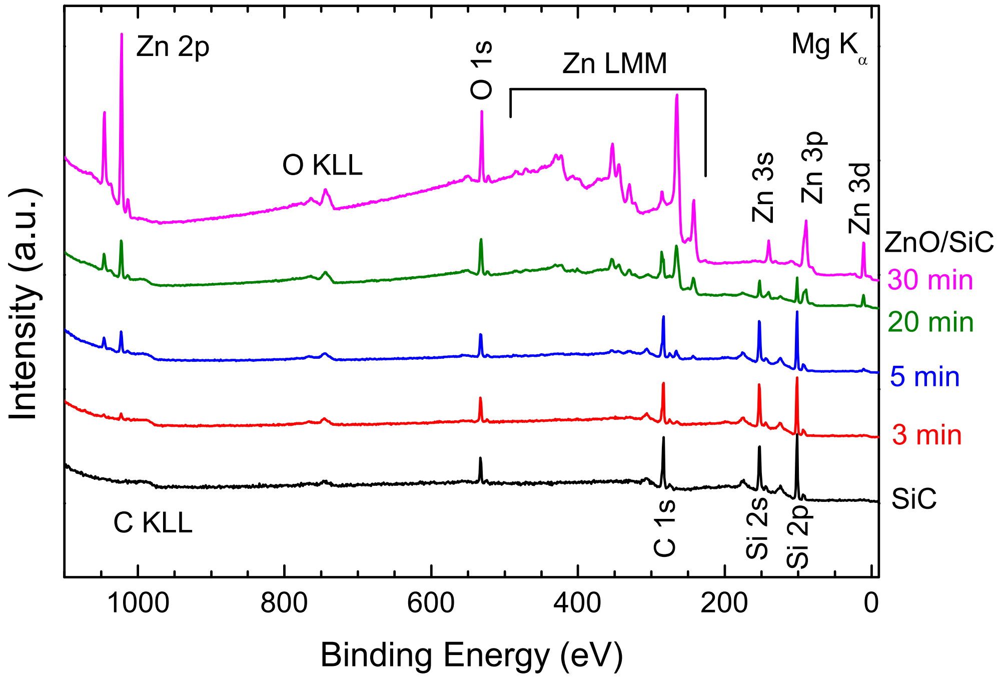

S.I.-1, Mg K_α excited XPS survey spectra of the cleaned SiC substrate and the ZnO/SiC thickness series. The spectra are vertically offset for clarity.

The C 1s XPS spectra of the cleaned SiC substrate and the ZnO/SiC thickness series prepared by MBE with varying deposition time are shown in S.I.-2. For easy comparison, each spectrum was normalized in such way that the background is one. The spectra consist of at least two features: one at about 283.7 eV is assigned to C-Si bonds, and the other one at around 285.8 eV is associated with C-O bonds (e.g., originated from surface contaminants). The intensity of C-Si feature gradually decreases with increasing ZnO deposition time, due to attenuation by the ZnO layer. Furthermore, the energetic position of C-Si feature shifts about -0.2 eV from the cleaned SiC to 20 min ZnO/SiC sample, as shown in the inset. Such shifting is consistent with that of Si features, as shown in Fig. 1(c). This further confirms that the shifting of Si features is indeed due to the interface-induced band bending.

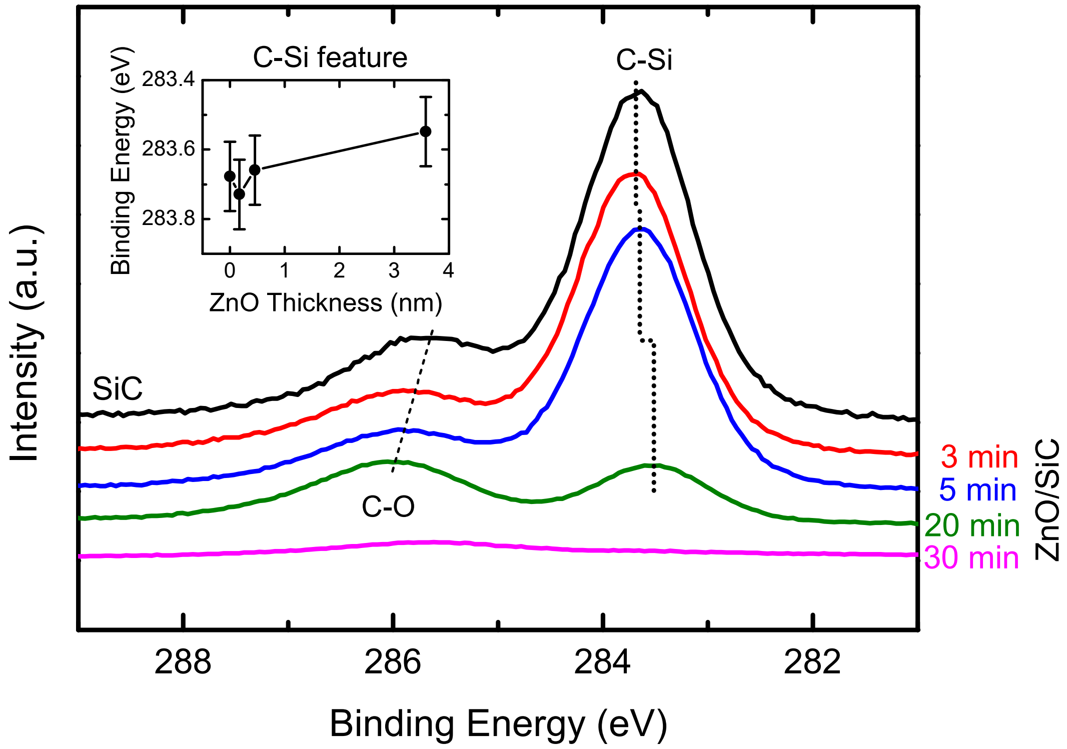

S.I.-2, C 1s spectra of the cleaned SiC substrate and the ZnO/SiC samples. Each spectrum was normalized in such way that the background is 1. The energetic position of C-Si feature is shown in the inset.

The Zn $2p_{3/2}$ XPS and Zn LMM XAES spectra of the ZnO/SiC thickness series prepared by MBE with varying deposition time are shown in S.I.-3. For easy comparison, each spectrum was normalized in such way that the background is zero and the maximum of main feature is one, and the spectra were shifted in energy scale to align the center of the main feature. The FWHM of Zn $2p_{3/2}$ lines and Zn $L_3M_{45}M_{45}$ (LMM) features of 20 and 30 min ZnO/SiC samples are larger than that of 5 min ZnO/SiC sample. This is likely an indication for the existence of an electron gas resulting in additional screening of the core hole and to inelastic scattering of photoelectrons because of excitation of plasmons. The broad Zn LMM spectrum of the sample with the thinnest ZnO can likely be attributed to a less defined material with different bond length and angles and limited long-range order, as well as quantum-size effects.

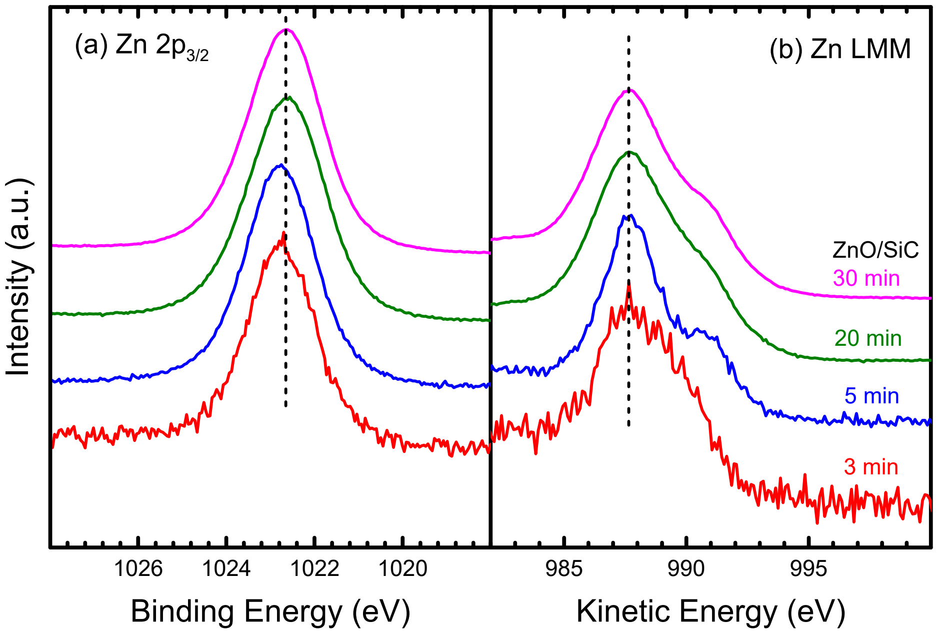

S.I.-3, Mg K_α excited (a) Zn $2p_{3/2}$ XPS and (b) Zn LMM XAES spectra of ZnO/SiC samples. Each spectrum was normalized in such way that the background is 0 and maximum of spectral intensity is 1. Note: the spectra were shifted in energy scale to align the center of peaks and offset vertically for clarity.
